# Sulfated Polysaccharides from Macroalgae Are Potent Dual Inhibitors of Human ATP-Hydrolyzing Ectonucleotidases NPP1 and CD39

**DOI:** 10.3390/md19020051

**Published:** 2021-01-22

**Authors:** Vittoria Lopez, Laura Schäkel, H. J. Maximilian Schuh, Michael S. Schmidt, Salahuddin Mirza, Christian Renn, Julie Pelletier, Sang-Yong Lee, Jean Sévigny, Susanne Alban, Gerd Bendas, Christa E. Müller

**Affiliations:** 1 Pharmaceutical & Medicinal Chemistry, Pharmaceutical Institute, University of Bonn, An der Immenburg 4, 53121 Bonn, Germany; vlopez@uni-bonn.de (V.L.); laura.schaekel@uni-bonn.de (L.S.); salahuddinmirza@gmail.com (S.M.); christian.renn@posteo.de (C.R.); s6saleee@uni-bonn.de (S.-Y.L.); 2 PharmaCenter Bonn, University of Bonn, An der Immenburg 4, 53121 Bonn, Germany; 3 Pharmaceutical & Cell Biological Chemistry, Pharmaceutical Institute, University of Bonn, An der Immenburg 4, 53121 Bonn, Germany; maxschuh@uni-bonn.de (H.J.M.S.); michael.sebastian.schmidt@uni-bonn.de (M.S.S.); gbendas@uni-bonn.de (G.B.); 4 Centre de Recherche du CHU de Québec—Université Laval, Québec City, QC G1V 4G2, Canada; julie.pelletier@crchudequebec.ulaval.ca (J.P.); jean.Sevigny@crchudequebec.ulaval.ca (J.S.); 5 Départment de Microbiologie-Infectiologie et d’Immunologie, Faculté de Médecine, Université Laval, Quebec City, QC G1V 0A6, Canada; 6 Pharmaceutical Institute, Christian-Albrechts-University of Kiel, Gutenbergstraße 76, 24118 Kiel, Germany; salban@pharmazie.uni-kiel.de

**Keywords:** adenosine, CD39, ectonucleotidase inhibitors, fucoidan, immuno-oncology, NPP1, NTPDase1, macroalgae constituents, sulfated polysaccharides

## Abstract

Extracellular ATP mediates proinflammatory and antiproliferative effects via activation of P2 nucleotide receptors. In contrast, its metabolite, the nucleoside adenosine, is strongly immunosuppressive and enhances tumor proliferation and metastasis. The conversion of ATP to adenosine is catalyzed by ectonucleotidases, which are expressed on immune cells and typically upregulated on tumor cells. In the present study, we identified sulfopolysaccharides from brown and red sea algae to act as potent dual inhibitors of the main ATP-hydrolyzing ectoenzymes, ectonucleotide pyrophosphatase/phosphodiesterase-1 (NPP1) and ecto-nucleoside triphosphate diphosphohydrolase-1 (NTPDase1, CD39), showing nano- to picomolar potency and displaying a non-competitive mechanism of inhibition. We showed that one of the sulfopolysaccharides tested as a representative example reduced adenosine formation at the surface of the human glioblastoma cell line U87 in a concentration-dependent manner. These natural products represent the most potent inhibitors of extracellular ATP hydrolysis known to date and have potential as novel therapeutics for the immunotherapy of cancer.

## 1. Introduction

**Figure 1:**
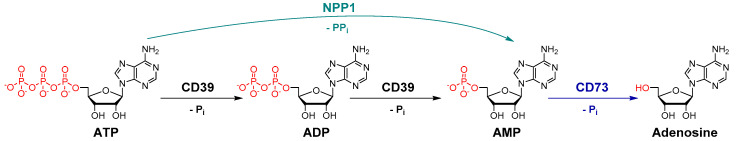
Hydrolysis of ATP to adenosine catalyzed by the key ectonucleotidases NPP1, CD39 and CD73 (P_i_, inorganic phosphate).

Nucleosides and nucleotides, e.g., adenosine, ATP and ADP, act as extracellular signaling molecules activating P1 (adenosine) or P2 (nucleotide) receptors [1,2,3,4,5]. Adenosine receptors are G protein-coupled receptors (GPCRs), while P2 receptors are further subdivided into P2Y (GPCRs) and P2X (ligand-gated ion channel) receptor subtypes. Nucleotides can be converted by a cascade of ectonucleotidases to adenosine (see Figure 1). Many tumor cells overexpress ectonucleotidases that metabolize proinflammatory ATP to adenosine, which exerts immunosuppressive, angiogenic, prometastatic and tumor growth promoting activities [5]. Ectonucleotidase inhibition has therefore been proposed as a novel approach for cancer immunotherapy [6,7]. Different classes of ectonucleotidases exist including nucleotide pyrophosphatase/phosphodiesterases (NPPs), nucleoside triphosphate diphosphohydrolases (NTPDases), alkaline phosphatases (AP) and ecto-5′-nucleotidase (cluster of differentiation 73 (CD73)). The NPP family consists of seven structurally related ectoenzymes (NPP1-NPP7), four of which are known to hydrolyze extracellular nucleotides [1,2,8]. NPP1 (CD203a, also known as PC-1 (plasma cell antigen-1), EC 3.1.4.1) and NPP3 catalyze the hydrolysis of a variety of nucleotides including nucleoside triphosphates (e.g., ATP and UTP), dinucleotide polyphosphates (e.g., Ap_3_A and Ap_4_A), cyclic dinucleotides (e.g., 2′,3″-cGAMP) and nucleotide sugars (e.g., UDP-glucose and ADP-ribose) [9,10,11,12], NPP1 preferentially hydrolyzes ATP into AMP and PPi. NPP4 was reported to hydrolyze the physiological dinucleotides Ap_3_A and Ap_4_A, while ATP hydrolysis by this enzyme is negligible [11]. NPP5 has recently been reported to hydrolyze nicotinamide adenine dinucleotide (NAD^+^) [13]. Other members of the NPP family, i.e., NPP2 (autotaxin), NPP6 and NPP7 (alkaline sphingomyelinase) act as phospholipases [8]. The NTPDase family comprises eight members (NTPDase 1-8), NTPDase1 (CD39, EC 3.6.1.5) being the most prominent member of this family in blood vessels and at the surface of leukocytes. They catalyze the hydrolysis of extracellular nucleoside tri- and diphosphates by cleaving off phosphate [8]. Ecto-5′-nucleotidase (CD73) hydrolyzes nucleoside monophosphates, e.g., AMP, to the corresponding nucleoside, e.g., adenosine. Thus, proinflammatory, antiproliferative ATP can be converted by subsequent action of NPP1 or CD39 and CD73 to immunosuppressive adenosine (Figure 1), which then accumulates in the tumor environment leading to immune escape of the tumor.

Recently, there has been an enormous interest in identifying and developing ectonucleotidase inhibitors, antibodies and small molecules, as novel cancer immunotherapeutics [4,6,7].

Several antibodies and small molecules have been developed for blocking the AMP-hydrolyzing, adenosine-producing ectoenzyme CD73, and the first drugs are currently evaluated in clinical trials for the immunotherapy of cancer to prevent the formation of immunosuppressive adenosine [14]. The blockade of enzymes that hydrolyze extracellular ATP to AMP could be even more efficient since it would lead to the accumulation of antiproliferative ATP in addition to preventing the formation of immunosuppressive adenosine due to the depletion of AMP as a CD73 substrate.

On the other hand, ATP, which acts on different subtypes of P2Y (G protein-coupled) and P2X (ligand-gated ion channel) receptors that are activated by different ATP concentrations depending on the receptor subtype, can also display proinflammatory effects since they belong to the danger-associated molecular patterns (DAMPs) [15]. In these cases, inhibition of ATP hydrolysis may result in proinflammatory effects.

**Figure 2:**
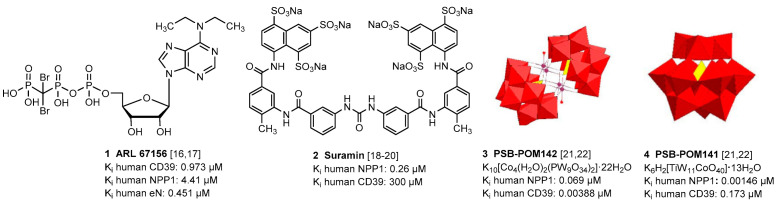
Selected inhibitors of ectonucleotidases NPP1 and CD39.

So far, only moderately potent and/or non-selective NPP1 and CD39 inhibitors have been described, which can be divided into nucleotides and non-nucleotides. *N*^6^-Diethyl-β,γ-dibromomethylene-ATP (ARL 67156, **1**) is a weak dual CD39/CD73 inhibitor with low metabolic stability [16,17]. Several other negatively charged compound classes including (poly)sulfonates such as suramin (**2**) and sulfoanthraquinones [18,19,20], and polyoxometalates (POMs), e.g., [TiW_11_CoO_40_]^8−^ (**3**) and [Co_4_(H_2_O)_2_(PW_9_O_34_)_2_]^10−^ (**4**) [21,22], were found to inhibit CD39 and/or NPP1. A selection of the most potent NPP1 and CD39 inhibitors is depicted in Figure 2.

Sulfated polysaccharides from sea algae, in particular fucoidans present in brown algae, e.g., from *Sargassum polycystum* [23], have been reported to possess antitumor activity in vitro and in vivo [24,25,26,27]. These natural products were shown to inhibit cell growth directly, e.g., by inducing apoptosis, and, in addition, to activate the immune system in its fight against cancer [28,29,30]. Their molecular mechanism of action is not fully understood at present, and the molecular targets are largely unknown [31]. This prompted us to study exemplary polysaccharides from red and brown algae, which are negatively charged like reported ectonucleotidase inhibitors (Figure 2), for inhibitory effects on these enzymes.

## 2. Results and Discussion

Four sulfated polysaccharides **5**–**8** (Table 1), extracted from different algae species, were investigated in the present study as potential inhibitors of ectonucleotidases. Compounds **5** and **8** represent sulfated xylogalactans from red algae, compounds **6** and **7** are brown algae-derived fucoidans. They were extracted, purified and chemically characterized as previously described [32,33,34]. The chemical characteristics of the used batches are shown in Table 1 and Table 2.

**Figure 3:**
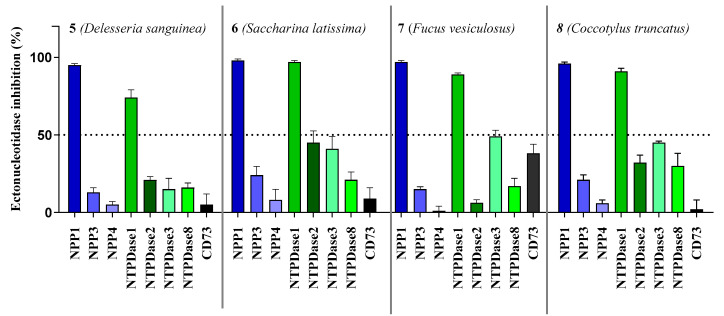
Ectonucleotidase inhibition by sulfopolysaccharides **5**–**8** extracted from sea algae. Compounds were tested at a concentration of 20,000 ng/mL. NPP1 and NTPDase2, 3 and 8 activities were analyzed using a CE-based assay employing ATP as a substrate as described in the experimental section; NPP3 was investigated using *p*-Nph-5′-TMP as a substrate (see Section 3.2 for details); NPP4 was tested with Ap_4_A as a substrate using a luciferase assay (for details see Section 3.3); NTPDase1 (CD39) activity was determined with a malachite green assay, or a CE-based assay, respectively (see Section 3.4 for details). The data is normalized with respect to positive (100%) and negative (0%) controls.

We initially tested the effects of the compounds at a concentration of 20,000 ng/mL on the most prominent ectonucleotidase families, NPPs (NPP1, 3, 4 and 5), NTPDases (NTPDase1, 2, 3 and 8) and CD73 (see Figure 3).

**Figure 4:**
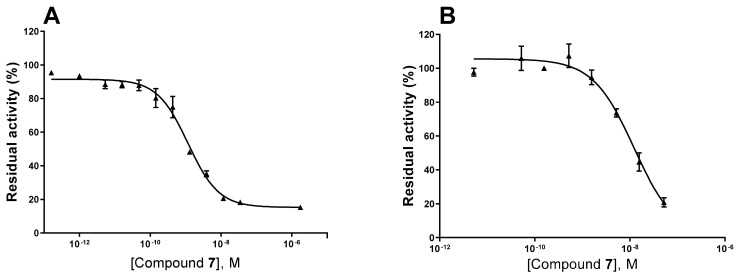
Concentration–inhibition curves of compound **7** at human NPP1 (**A**) and human CD39 (**B**). For details see Experimental Section.

All compounds showed the highest inhibition of NPP1, which was completely inhibited at the test concentration, combined with high selectivity versus the other NPP subtypes. The second highest inhibition was observed at NTPDase1 (CD39) while inhibition of all other enzymes was below 50%. As a next step, we investigated concentration-dependent inhibition by determining full concentration–inhibition curves for all four compounds at NPP1 and CD39, and determined the compounds’ K_i_ values (see Table 3). Curves for compound **7** as a representative inhibitor are depicted in Figure 4. Compound **7** was selected because it showed the lowest molecular weight of all tested sulfated polysaccharides and was available in high quantity.

**Figure 5:**
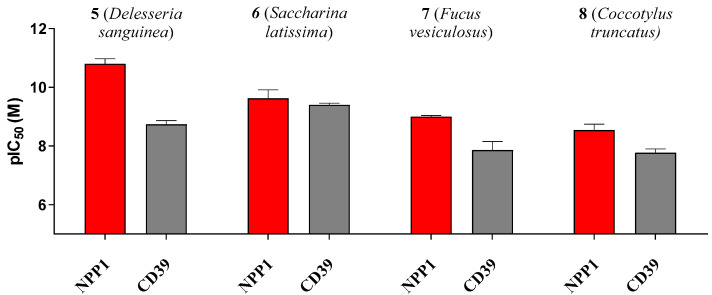
*p*IC_50_ (-log IC_50_) values of compounds **5**–**8** at the ectonucleotidases NPP1 and CD39 are shown. Error bars represent SD values. For IC_50_ values see Table 3.

Some of the investigated sulfated polysaccharides from sea algae were discovered to be the most potent NPP1 and CD39 inhibitors described to date displaying nano- to subnanomolar potencies. The potency calculated in nmol/L (nM) did not depend on the molecular weight. Sulfated polysaccharide **5** (MW 214 kDa) was almost 3-fold more potent at NPP1 as compared to compound **6** (MW 534 kDa). Compound **5** showed 33-fold selectivity for NPP1 over CD39, while **6** was about equipotent. Compounds **7** and **8** displayed somewhat lower potency combined with moderate selectivity for NPP1 versus CD39 (see Figure 5). Compound **5** is an extremely potent NPP1 inhibitor with picomolar inhibitory potency at NPP1 (K_i_ 0.0517 nM) showing ancillary CD39 inhibition (K_i_ 1.72 nM), but being highly selective versus all other investigated ectonucleotidases. Sulfated polysaccharide **6** is about equipotent at both NPP1 and CD39, and can be envisaged as a dual NPP1/CD39 inhibitor. Such dual activity could be a big advantage in cancer therapy since both ATP-hydrolyzing enzymes may be upregulated and constitute redundant pathways for nucleotide degradation.

**Figure 6:**
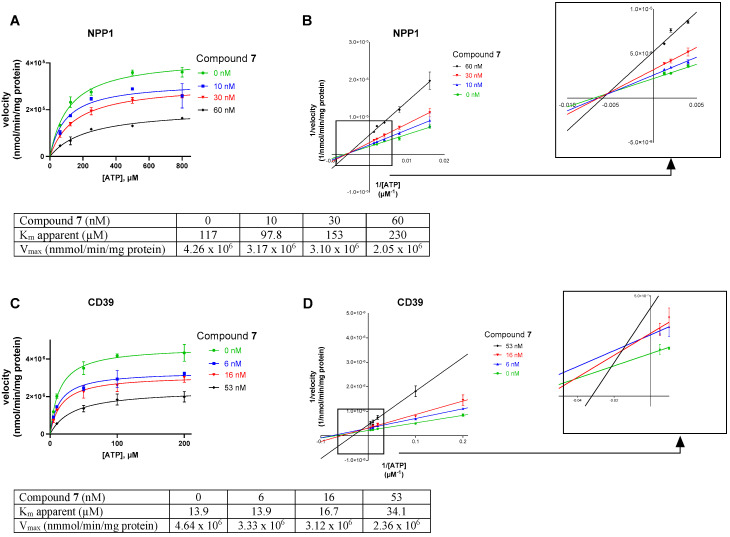
Investigation of the enzyme inhibition type for inhibitor **7** at NPP1 (**A**,**B**) and CD39 (**C**,**D**). In (**A**,**C**) the Michaelis–Menten curves are shown without and in the presence of different concentrations of inhibitor **7**. For the determination of the inhibition type, Lineweaver–Burk plots are shown in (**B**,**D**) (see Section 3.1 and Section 3.4 for experimental details). The results indicate a non-competitive/mixed type of inhibition. The kinetic parameters of ATP hydrolysis by CD39 and NPP1, respectively, in the absence and presence of inhibitor **7** are provided.

Next, we studied the inhibition type for compound **7**, the compound with the lowest molecular weight, as a representative for this new class of ectonucleotidase inhibitors using ATP as a substrate. Considering the size of the compounds (with molecular masses ranging from 38 to 534 kDa) and based on their structures, we presumed an allosteric mechanism of inhibition. The effect of inhibitor **7** on the kinetics of NPP1 and CD39 was investigated. In both cases, the Michaelis–Menten plots (Figure 6A,C) clearly showed a decrease in *V_max_* in the presence of the inhibitor, which is indicative of a non-competitive or mixed type of inhibition. Moreover, the apparent K_m_ values are increased at higher inhibitor concentrations. The Lineweaver–Burk plot [39] confirmed a non-competitive/mixed type of inhibition (Figure 6B,D) at both enzymes, NPP1 and CD39.

**Figure 7:**
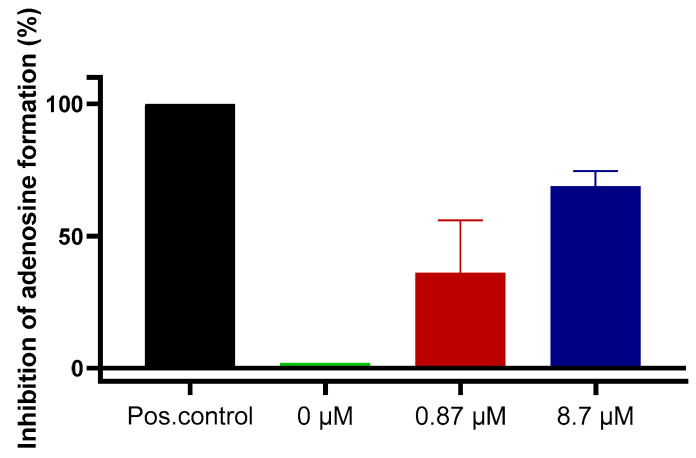
Blockade of extracellular adenosine formation from ATP by compound **7** on human U87 glioblastoma cells. The cells were treated with ATP (ectonucleotidase substrate) and incubated for 3 h in the presence of dipyridamole to prevent cellular uptake of adenosine, and EHNA to prevent adenosine deamination. Adenosine was quantified by CE-UV. Results represent means of two independent experiments each performed in quadruplicate measurements. Positive (Pos.) control: the potent NPP1 inhibitor PSB-POM141 (**4**, 10 µM) or the potent CD73 inhibitor PSB-19316 (0.1 µM) were added both of which led to a full blockade of NPP1 and CD73 activity thereby preventing the formation of adenosine. For details see Experimental Section 3.6.

Finally, we investigated compound **7** in a more complex cellular system. The human glioblastoma cell line U87 had previously been shown to express NPP1 and CD73 [40,41,42,43], but only negligible amounts of CD39 [44]. These enzymes are known to convert ATP to adenosine in a sequential reaction cascade (see Figure 1). Therefore, we used this cancer cell line to determine the hydrolysis of extracellular ATP, added to the cells, resulting in the formation of adenosine, which is known to produce antiproliferative, antiangiogenic, metastasis-promoting and immunosuppressive effects when released into the tumor microenvironment. Dipyridamole was added to block the cellular uptake of adenosine via the SLC29 transporter family [45], and erythro-9-(2-hydroxy-3-nonyl)adenine (EHNA) was present to inhibit metabolism of adenosine to inosine [46]. Cells treated with ATP, dipyridamole and EHNA were studied in the absence and in the presence of sulfopolysaccharide **7**, and extracellular adenosine accumulation was quantified by capillary electrophoresis coupled to UV detection. The results clearly showed a concentration-dependent inhibition of adenosine formation by sulfopolysaccharide **7** in U87 glioma cells, which is assumed to be due to the blockade of NPP1 by inhibitor **7**. These data confirmed the strong inhibitory activity of ectonucleotidase inhibitor **7** (as a representative of the chemical class of sulfopolysaccharides) on the formation of extracellular adenosine from ATP by a human glioblastoma cell line (Figure 7).

## 3. Materials and Methods

The sulfated polysaccharides **5**–**8** were isolated from the two brown algae *Saccharina latissima* (Laminariales, Laminariaceae) (North Atlantic Faroe Island) and *Fucus vesiculosus* (Fucales, Fucaceae) and the two red algae *Delesseria sanguinea* (Ceramiales, Delesseriaceae) and *Coccotylus truncatus* (Gigartinales, Phyllophoraceae) (both collected in the sublittoral habitat of the large artificial reef near Nienhagen (Baltic Sea, Germany). They were extracted, purified and chemically characterized as previously described [32,33,34], whereby compound **7**, fucoidan from *Fucus vesiculosus* was purchased from Sigma-Aldrich, catalog No. F5631, Lot No SLBC4004 V). In short: the pulverized algal material was defatted with Soxhlet extraction (99% *v/v* ethanol). The main extraction was performed with demineralized water at 85 °C for 8 h (compound **5**) and aqueous 2% CaCl_2_ at 85 °C for 2 h (compounds **6** and **8**) (reflux condition). The supernatant was evaporated and precipitated with ethanol (final concentration 60% *v*/*v*) at 4 °C. Further steps involved centrifugation, dissolving in demineralized water, dialysis and lyophilization. The analyzed chemical parameters included sulfate content and degree of sulfation, weight average molar mass, monosaccharide composition, contents of protein and uronic acids.

### 3.1. Chemicals

Nucleotides (AMP, ADP, ATP and cAMP), nucleosides (adenosine, inosine and uridine), dipyridamole, erythro-9-(2-hydroxy-3-nonyl)adenine (EHNA), disodium hydrogenphosphate and sodium dodecyl sulfate were purchased from Merck KGaA (Darmstadt, Germany). Sodium chloride, potassium chloride, potassium dihydrogen phosphate, sodium hydrogencarbonate, D-glucose, *N*-[2-hydroxyethyl]piperazine-*N*’-[2-ethanesulfonic acid] (HEPES), calcium chloride and magnesium sulfate were purchased from PAN Biotech GmbH (Aidenbach, Germany). Dulbecco’s modified Eagle medium, fetal calf serum (FCS), penicillin/streptomycin (P/S) and L-glutamine were also from PAN Biotech GmbH.

### 3.2. NPP1 Assay

Recombinant soluble human NPP1 was obtained as previously described [22]. Compounds **5**–**8** were tested as inhibitors of human NPP1 at a concentration of 20,000 ng/mL vs. ATP (400 µM) as a substrate. Subsequent concentration–inhibition curves were performed using several dilutions of the test compounds prepared in assay buffer (10 mM *N*-cyclohexyl-2-aminoethanesulfonic acid (CHES), 2 mM CaCl_2_ and 1 mM MgCl_2_, pH 9.00). The human NPP1, prepared as previously described [22], was diluted in the assay buffer, and 0.224 μg of the enzyme were employed per vial. The mixture was incubated for 30 min at 37 °C, and the reaction was terminated by heating at 90 °C for 5 min. After cooling down on ice, analysis was carried out using capillary electrophoresis (CE). Positive and negative controls were studied in parallel. Data collection and peak area analysis were performed by the P/ACE MDQ software 32 KARAT obtained from Beckman Coulter (Fullerton, CA, USA). A polyacrylamide-coated capillary was used (30 cm (20 cm effective length) × 50 μm (id) × 360 μm (od) purchased from Chromatographie Service GmbH (Langerwehe, Germany)). Samples were injected electrokinetically by applying a voltage of −6 kV for 30 s. Finally, analytes were separated by applying a separation voltage of −20 kV, and detected by UV at 260 nm. The *IC_50_* values were determined by nonlinear curve fitting using the GraphPad Prism software 7.0. The mechanism of inhibition of human NPP1 was determined by employing different concentrations of the inhibitor (0, 10, 30 and 60 nM) vs. five different substrate concentrations ranging from 62 to 800 μM ATP. The assay procedure and operation conditions were the same as described above. The experiments were conducted two times, each in triplicates. A Lineweaver–Burk was calculated using GraphPad Prism 7.0 for predicting the inhibition type of the inhibitor.

### 3.3. NPP3 Assay

Recombinant soluble human NPP3 was obtained as previously described [47]. Compounds **5**–**8** were screened at a concentration of 20,000 ng/mL vs. 400 μM *p*-Nph-5′-TMP to study a potential inhibition of NPP3, using a colorimetric assay as previously described [38]. Soluble, purified human NPP3 was diluted in the assay buffer (50 mM TRIS HCl, 2 mM CaCl_2_ and 0.2 mM ZnCl_2_ pH 9.00), and 0.45 µg of enzyme per vial was used. The mixture was incubated for 30 min at 37 °C, and the reactions were subsequently terminated by adding 20 μL of 1.0 N NaOH. The assay is based on the enzymatic ester hydrolysis of *p*-Nph-5′-TMP that results in the formation of *p*-nitrophenolate. The absorption maximum was measured at 400 nm using a BMG PheraStar FS plate reader (BMG Labtech GmbH, Ortenberg, Germany). Each analysis was repeated three times in triplicate measurements.

### 3.4. NPP4 Assay

Recombinant human soluble NPP4 was obtained as previously described [48]. Screening of the test compounds **5**–**8** was performed at a concentration of 20,000 ng/mL versus 20 μM of Ap_4_A as a substrate employing a previously described luminescence-based assay [48]. The assay buffer consisted of 10 mM HEPES, 1 mM MgCl_2_ and 2 mM CaCl_2_ (pH 8.0). The enzyme reaction was started by adding 0.14 μg of human purified NPP4 to the reaction mixture which was incubated at 37 °C for 60 min. The released product, ATP, was quantified using the luciferin–luciferase reaction. The firefly luciferase reacts with D-luciferin in the presence of the formed ATP and Mg^2+^, which act as cofactors. The resulting luminescence correlates to the enzyme activity. It was measured at 560 nm using a microplate reader (BMG PheraStar, Labtech GmbH, Ortenberg, Baden-Württemberg, Germany). Three independent experiments were performed, each in duplicates with positive and negative controls studied in parallel.

### 3.5. NTPDase Assays

Human NTPDases (subtypes 1, 2, 3 and 8) were recombinantly expressed as previously described and cell membrane preparations overexpressing one of the NTPDase subtypes were used for the experiments according to described procedures [46]. For monitoring the inhibitory activity of the compounds at the NTPDases two different assay system were utilized. The malachite green assay was used for the determination of NTPDase1 (CD39) concentration–inhibition curves with compound **5**–**7** and for the determination of the inhibition type of compound **7**. The CE-based assay was utilized for selectivity studies of compound **5**–**8** at NTPDase2, NTPDase3 and NTPDase8, and for the determination of concentration–inhibition curves for compound **8** at human CD39. The test compounds were investigated at 20,000 ng/mL for initial screening, and subsequently at different concentrations for obtaining concentration–inhibition curves. The enzyme amounts were selected after enzymatic titration and adjusted to ensure a conversion rate of 10–20%; for NTPDase1 assays, 0.1 µg of human umbilical cord membrane preparation [49,50] was employed per vial, for NTPDase2 assays, 0.07 µg of recombinant enzyme was used, for NTPDase3 assays, 0.45 µg of enzyme was employed and for NTPDase8 assays, 0.52 µg of enzyme was utilized. Human NTPDase1, -2, -3 and -8 were expressed as previously described [49,50]. When the malachite assay was used, the reaction was initiated by the addition of 50 µM ATP (*K_m_* (NTPDase1) = 17 µM) [50]. The reaction buffer consisted of 10 mM HEPES, 2 mM CaCl_2_ and 1 mM MgCl_2_, pH 7.4. The enzyme reaction mixture was incubated at 37 °C for 15 min. The released phosphate was quantified by adding malachite-green and molybdate reagents, incubating for 20 min at room temperature, and measuring the UV-absorption at 600 nm [51]. The prediction of the mechanism of inhibition at human NTPDase1 was determined for compound **7** as a representative example employing different concentrations of the investigated inhibitor (0, 6, 16 and 53 nM of **7**) vs. five different substrate concentrations of ATP (from 5 to 200 µM). The assay procedure and operation conditions were the same as described above. A Lineweaver–Burk plot was calculated using GraphPad Prism 7.0 for predicting the inhibition type of the compound. In the CE assay, the selected substrate concentration of ATP was 100 µM for human NTPDase2, -3 and -8. The reaction buffer was the same as for NTPDase1 described above. The mixtures of enzyme with the substrate and test compound were incubated at 37 °C for 30 min, and the enzymatic reaction was stopped by heating it for 10 min at 95 °C. The released products were separated by capillary electrophoresis and quantified by their UV-absorption at 260 nm in the presence of ADP and AMP as external standards as previously described [48]. Positive and negative controls were studied in parallel.

### 3.6. CD73 Assay

Soluble human CD73 was recombinantly expressed and purified as described [52], and the assays were performed according to a described procedure [53]. The enzymatic reaction was performed by mixing 0.36 ng of human CD73 [52], with the test compound (20,000 ng/mL) in the assay buffer (25 mM Tris, 140 mM sodium chloride and 25 mM sodium dihydrogenphosphate, pH 7.4), and 5 µM of [2,8-^3^H]AMP (specific activity 7.4 × 10^8^ Bq/mmol, 20 mCi/mmol, American Radio-labeled Chemicals, MO, USA, distributed by Hartmann Analytic, Germany) was added. The enzymatic reaction was performed for 25 min at 37 °C in a shaking water bath. Then, 500 µL of cold precipitation buffer (100 mM lanthanum chloride and 100 mM sodium acetate, pH 4.00) was added to stop the reaction and to facilitate precipitation of free phosphate and unconverted [2,8-^3^H]AMP. After the precipitation was completed (after at least 30 min on ice), the mixture was separated by filtration through GF/B glass fiber filters using a cell harvester (M-48, Brandel, MD, USA). After washing each reaction vial three times with 400 µL of cold (4 °C) demineralized water, 5 mL of the scintillation cocktail (ULTIMA Gold XR, PerkinElmer, MA, USA) was added, and the radioactivity was measured by scintillation counting (TRICARB 2900 TR, Packard/PerkinElmer; counting efficacy: 49–52%). Positive and negative controls were tested in parallel.

### 3.7. Experiments on U87 Glioblastoma Cells

U87 glioblastoma cells were grown in cell culture flasks until reaching a confluent cell layer. Cells were cultivated at 37 °C and 5% CO_2_ in Dulbecco’s modified Eagle medium (DMEM) with additions of 10% FCS, 1% penicillin/streptomycin and 1% L-glutamine. The cells were rinsed with phosphate-buffered saline (PBS) and detached with ethylenediaminetetraacetic acid (EDTA). Thereafter, they were counted in a cell counter (CASY^®^ 1 Model TT, Schärfe System GmbH, Reutlingen, Germany), then rinsed three times with PBS before they were resuspended in Krebs-HEPES buffer at a concentration of 1×10^6^ cells/mL per well [54]. Cells (1 mL of a cell suspension containing 10^6^ cells) were then pipetted into each well of a 24-well plate (Sarstedt AG, Nümbrecht, Germany). Subsequently, the cells were pretreated with dipyridamole (20 µM) for 30 min to block nucleoside transport. Adenosine deaminase (ADA) activity was inhibited by the addition of 1 µM of the inhibitor EHNA, which was added to each sample, and controls [4]. Thereafter, inhibitor **7** was added, and the samples were incubated with 300 µM of ATP for 3 h. Then, an aliquot of 230 µL of solution from each vial was transferred to 1.5 mL Eppendorf tubes, and heated at 95 °C for 10 min to inactivate the enzymes in order to avoid further nucleotide degradation. Before performing capillary electrophoresis (CE) measurements, the samples were centrifuged at 600 × *g* to remove insoluble material like cell debris from the supernatant. Until CE measurement, the supernatants were stored at −20 °C. Nucleotides (ATP, ADP, AMP and cAMP) and nucleosides (adenosine, inosine and uridine), which were also used as standards to monitor efficient separation by CE, were dissolved in Krebs-HEPES buffer at a concentration of 10 µM [54] and stored at −20 °C as well. For analysis of the formed products, CE measurements were performed according to published procedures [55].

## 4. Conclusions

In conclusion, we identified sulfopolysaccharides isolated from sea algae to act as extremely potent, noncompetitive inhibitors of the ectonucleotidases NPP1 and CD39 with somewhat higher inhibitory potency against NPP1. Both of these enzymes catalyze the hydrolysis of proinflammatory, antiproliferative ATP yielding, in concert with the AMP-hydrolyzing ectoenzyme CD73, adenosine, which displays immunosuppressive and tumor-promoting activities. In fact, we observed reduced formation of adenosine in human glioblastoma U87 cells treated with a representative example of the investigated sulfopolysaccharides. These effects might explain or at least contribute to the previously observed anticancer effects of sulfated algae polysaccharides from sea weeds, which were shown to be non-toxic and well tolerated by humans. The sulfate groups are believed to be essential for their ectonucleotidase-inhibitory effects because other negatively charged compounds, e.g., some polyoxometalates, can also inhibit these ATP-hydrolyzing enzymes with high potency. The substrate ATP itself is also a negatively charged compound, and the enzymes may therefore preferably recognize negatively charged molecules. While ATP would subsequently be guided to the substrate binding pocket, the large anionic polymers and cluster compounds likely remain attached to the initial recognition site at the surface of the enzymes showing allosteric inhibition. In future studies, we plan to investigate the role of the sulfate groups on the seaweed polysaccharides for their enzyme-inhibitory potency by investigating derivatives without or with lower degrees of sulfation.

## Figures and Tables

**Table 1:** Basic characteristics of investigated sulfated algae polysaccharides.

Compound	Type of Sulfated Polysaccharide	Extracted Alga Species	Degree of Sulfation ^a^	Molar Mass (kDa) ^b^	Proteins (%) ^c^	Uronic Acids (%) ^d^
**5**	Xylogalactan	*Delesseria sanguinea*	0.65 ± 0.02	214 ± 28	7.24 ±0.07	3.96 ± 0.54
**6**	Fucoidan	*Saccharina latissima*	0.52 ± 0.01	534 ± 11	8.08 ±0.09	7.42 ± 0.18
**7**	Fucoidan	*Fucus vesiculosus*	0.59 ± 0.01	38 ± 1	7.07 ± 1.59	0.27 ± 0.27
**8**	Xylogalactan	*Coccotylus truncatus*	0.52 ± 0.01	128 ± 4	3.74 ±1.29	9.50 ± 0.23

^a^ Average number of sulfate groups per monosaccharide related to the total glycan content; it was calculated by means of the SO_3_Na, which was derived from % sulfur content determined by elemental analysis (mean ± SD, *n* = 2). ^b^ Mean weight average molar mass, determined by SEC-MALS-RI, mean ± SD (mean ± SD, *n* = 3). ^c^ The content of protein was calculated by elemental analysis (% nitrogen) (mean ± SD, *n* = 2). ^d^ Determined according to the method of Blumenkrantz et al. [35] and Filisetti-Cozzi et al. [36] (mean ± SD, *n* = 2 × 2).

**Table 2:** Composition of investigated sulfated algae polysaccharides.^a^

Compound	Type of Sulfated Polysaccharide	Fucose (mol%)	Galactose (mol%)	Xylose (mol%)	Mannose (mol%)	Glucose (mol%)	Rhamnose (mol%)
**5**	Xylogalactan	0.0	75.3	16.1	2.5	6.1	0.0
**6**	Fucoidan	53.4	13.6	6.7	5.4	19.8	1.3
**7**	Fucoidan	83.1	7.3	6.5	2.0	0.4	0.7
**8**	Xylogalactan	0.0	87.6	3.8	6.7	2.0	0.0

^a^ Determined according to the method of Blakeney et al. [37].

**Table 3:** Potencies of investigated compounds at ectonucleotidases NPP1 and CD39 in comparison to standard inhibitors.

Compound	Compound Nameor Algal Species	Human NPP1Ki ± SEM (nM)	Human CD39Ki ± SEM (nM)
**Standard ectonucleotidase inhibitors**			
**1**	ARL 67156	973 ± 239 [14] ^a^	4410 ± 3,530 [14] ^b^
**2**	Suramin	780 ± 81 [38] ^b^	300,000 ± 100 [18] ^b^
**3**	PSB-POM142	690 ± 4 [22] ^b^	3.88 ± 1.40 [22] ^c^
**4**	PSB-POM141	1.46 ± 0.01 [22] ^b^	173 ± 4 [22] ^c^
**Sulfated algae polysaccharides**			
**5**	*Delesseria sanguinea*	0.0517 ± 0.0016 ^b^ (11.1 ng/mL)	1.72 ± 0.00 ^d^ (366 ng/mL)
**6**	*Saccharina latissimi*	0.136 ± 0.001 ^b^ (72.8 ng/mL)	0.408 ± 0.001 ^d^ (218 ng/mL)
**7**	*Fucus vesiculosus*	1.19 ± 0.00 ^b^ (45.2 ng/mL)	12.3 ± 0.0 ^d^ (469 ng/mL)
**8**	*Coccotylus truncatus*	5.33 ± 0.00 ^b^ (682 ng/mL)	16.0 ± 0.0 ^b^ (2045 ng/mL)

^a^ absorbance-based assay; ^b^ CE-based assay; ^c^ fluorescence polarization (FP) assay; ^d^ malachite-green assay.

## Data Availability

The data presented in this study are available on request from the corresponding author. The data are not publicly available due to privacy reasons.

## Abbreviations

Ap_4_A	diadenosine tetraphosphate
CD	cluster of differentiation
CE	capillary electrophoresis
CHES	*N*-cyclohexyl-2-aminoethanesulfonic acid
CD39	nucleoside triphosphate diphosphohydrolase-1
CD73	ecto-5′-nucleotidase
DAD	diode array detector
DMEM	Dulbecco’s Modified Eagle Medium
EDTA	ethylenediaminetetraacetic acid
EHNA	erythro-9-(2-hydroxy-3-nonyl)-adenine
HEPES	4-(2-hydroxyethyl)piperazine-1-ethanesulfonic acid
NTPDase1	nucleoside triphosphate diphosphohydrolase-1
NPP1	nucleotide pyrophosphatase/phosphodiesterase-1 (CD203a)
pIC_50_	negative log of the half-maximal inhibitory concentration
PBS	phosphate-buffered saline
*p*NP-TMP	*p*-nitrophenyl thymidine 5-monophosphate
PSB	Pharmaceutical Sciences Bonn
SDS	sodium dodecylsulfate
